# The association of climate-induced stressors on risk of negative sentiment: An analysis from 462 million geotagged tweets in Europe

**DOI:** 10.1016/j.isci.2025.113933

**Published:** 2025-11-03

**Authors:** Tareq Al-Ahdal, Sandra Barman, Barrak Alahmad, Stella Dafka, Elisa Gallo, Joan Ballester, Mikhail Sofiev, Marina Romanello, Till Bärnighausen, Michael Gertz, Joacim Rocklöv

**Affiliations:** 1 Section for Oral Health, Heidelberg Institute of Global Health, Heidelberg University Hospital, Medical Faculty Heidelberg, Heidelberg University, Heidelberg, Germany; 2 Heidelberg Institute of Global Health (HIGH), Heidelberg University Hospital, Heidelberg University, Heidelberg, Germany; 3 Interdisciplinar Center for Scientific Computing (IWR), Heidelberg University, Heidelberg, Germany; 4 Bioeconomy and Health, RISE Research Institutes of Sweden, Gothenburg, Sweden; 5 Department of Environmental Health, Harvard T.H. Chan School of Public Health, Harvard University, Boston, MA, USA; 6 Department of Epidemiology and Global Health, Umeå University, Umeå, Sweden; 7 Institute of Computer Science, Heidelberg University, Heidelberg, Germany; 8 ISGlobal, Barcelona, Spain; 9 Finnish Meteorological Institute, Helsinki, Finland; 10 UCL Institute for Global Health, London, UK

**Keywords:** Earth sciences, Climatology, Social sciences, Psychology

## Abstract

This study examines how climate-induced health risks influence negative sentiments on European social tweets from 2015 to 2022. Analyzing over 400 million tweets using NLP tools (NLTK, LIWC22) and spatial-temporal aggregation at the NUTS2 weekly level, we applied a Poisson generalized additive model (GAM) with integrated nested Laplace approximation (INLA) and fused lasso regularization to capture sentiment fluctuations. Results show negative sentiments rise by up to 0.36% when maximum temperatures exceed 26.9°C and by 0.49% during severe droughts (SPI < −3.72). Elevated alder pollen counts (>135 grains/m^3^) also increase risk of negative sentiment by 0.21%, while temperatures below 2.9°C reduce it by 0.63%. No significant association was found with heat-related mortality or West Nile virus incidence. These findings suggest specific climate-related health factors—high temperatures, droughts, and pollen—trigger negative social media reactions, whereas others, such as mortality and infectious outbreaks, appear unnoticed in public sentiment.

## Highlights

• Climate stressors, including heat, drought, and pollen, drive negative sentiment on X
• Max temperatures >26.9°C and severe droughts increase negative sentiment risk by up to 0.49%
• Low temperatures (<2.9°C) reduce negative sentiment risk, while heat mortality shows no effect
• Over 400 million tweets analyzed reveal selective climate-health impacts on public sentiment

## Graphical abstract

**Figure:**
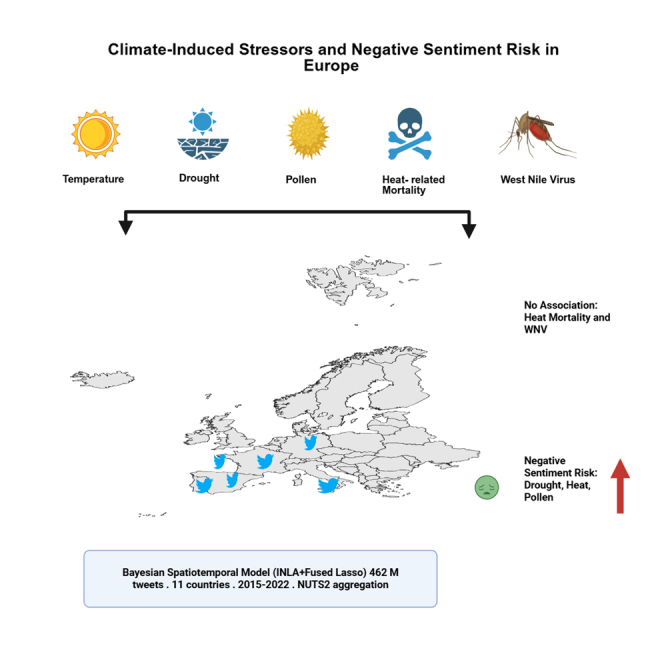

## Introduction

Our planet’s climate is undergoing substantial changes, resulting in changes in the occurrence of climatic hazards, such as, changes in seasonal weather patterns and more frequent extreme weather-related events, including extreme heat.^1^ Climatic hazards are known to increase the risk of adverse human health impacts, such as heat-related mortality, air-pollution, infectious diseases, and mental health.^2^^,^^3^ For example, higher pollen concentrations are commonly associated with increased rates of allergic reactions,^4^ high temperatures associate to heat-related mortality and increase the suitability of transmission of vector-borne diseases, such as West Nile virus (WNV).^5^ What is not so well understood is the systematic way of quantifying and monitoring the link that climate change can affect emotional reactions, leading to impacts on the mental health of individuals^6^ inducing anxiety, trauma, fatigue, and grief, and how environmental instability can lead to eco-anxiety, fear, and uncertainty.^7^

In the past ten years, there has been significant research in leveraging digital data from social media for public health studies, including infectious diseases,^8^ to monitor sentiment and behavioral responses during health crises. In the climate health domain, studies have utilized sentiment as a proxy for well-being.^9^ These studies analyze online sentiment expression in social media textual data using machine learning approaches^10^ and tools such as the Valence Aware Dictionary and sEntiment Reasoner (VADER)^11^ or dictionary-based methods, such as linguistic inquiry and word count (LIWC).^12^ These studies have opened new pathways to understanding trends of distress and wellbeing on a larger scale and in real-time.

Previous studies have assessed the relationships between climatic variables and sentiment from social media,^13^ suggesting that weather can influence, distress, give rise to negative emotions, and reduce well-being. Results consistently demonstrate that increases in temperature and precipitation are often associated with changes in sentiments.^14^ For instance, a comprehensive analysis from previous Lancet countdown indicators in 2021 and 2022, based on global Twitter (renamed X) data, revealed that heat waves worsened expressed sentiments in 2020. In addition, the Lancet countdown 2021 study mentioned a slight decrease in positive sentiment due to heat waves, while extreme precipitation days contributed to a pronounced reduction in positive expressions.^15^^,^^16^ A study from China found that extreme weather conditions worsen emotional responses to social media on the platform Weibo.^9^ Building on this evidence, recent research utilizing geotagged social media posts from the Weibo platform found extreme cold temperatures by one unit increase significantly decrease sentiments by approximately 0.161 (0.272) units.^17^ This growing body of literature suggests that weather-induced emotional responses captured through social media can serve as potential indicators of well-being of the populations represented in social media, offering insights into the broader societal impacts of climate distress. Due to the large availability of social media data, it may fill a gap in our understanding of the social impacts from climate hazards and the interconnections between climate change, distress and emotional well-being at a population level and across space and time in real time.^18^ Promoting well-being is a central objective of the Sustainable Development Goals established by the United Nations.^19^ In response, many national and municipal governments worldwide are increasingly integrating Subjective Well-Being (SWB)^20^ indicators into their policy frameworks, complementing traditional objective measures of development and economic progress. This shift underscores the importance of understanding the factors that influence well-being in the context of climate change.

While previous studies have examined the impact of temperature and precipitation on negative sentiments from social media, no studies to date have investigated how a wider range of climate-health related indicators associate to positive and negative sentiments. Consequently, this study aims to bridge this knowledge gap by leveraging data from the X platform along with a suite of climate-health indicators from the Lancet Countdown on Climate Change and Health in Europe report.^21^ Specifically, we aimed to study indicators of temperature, drought, heat-attributable mortality, pollen concentrations, and West Nile Fever. We analyzed the association of varying climate-induced stressors on negative sentiments using a formal Bayesian spatiotemporal modeling with fused lasso integration to provide a more precise understanding of the relationship between climate-health hazards to emotional reactions on X.

## Results

Descriptive statistics summarizing the distribution of tweets across languages and years are presented in the supplemental information in Tables S1 and S2, and Figure S4.

### Maximum temperature and negative sentiment risk

**Figure 1:**
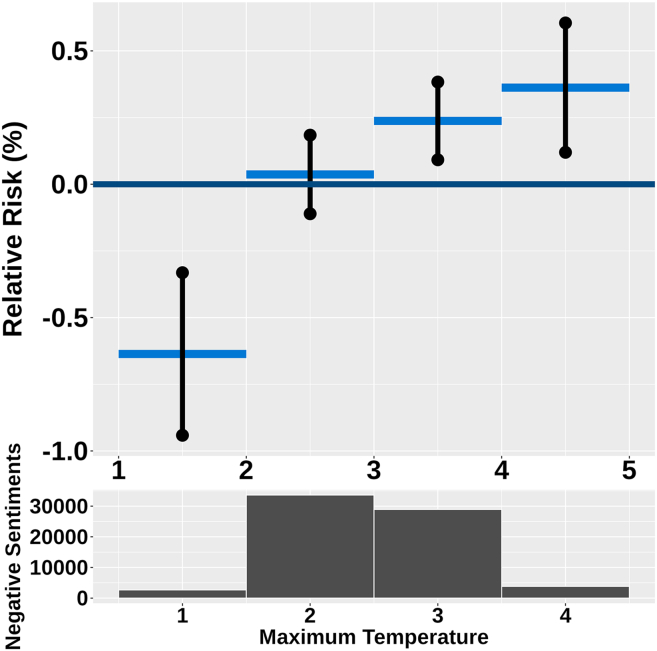
The non-linear random effect of weekly maximum temperature (dark blue graph) has been analyzed by categorizing the weekly maximum temperature in each region into 4 levels: low, mid, average, and high temperature The temperature categories are below 2.96°C, from 2.96°C to 14.9°C, from 14.9°C to 26.9°C, and above 26.9°C. The dark graph represents the upper and lower bounds. It shows that the effect is significantly below zero for the low-temperature level and significantly above zero for the high-temperature level. These upper and lower bounds are 95% significant bounds. The dark black histogram below shows the distribution of negative sentiments in each category.

Our findings indicate a clear relationship between maximum temperature and risk of negative sentiment (Figure 1). Statistical analysis reveals that the relative risk of negative sentiments also rises as maximum temperature increases. Notably, the highest relative risk is observed at maximum temperatures exceeding 26.9°C, with an increase or a change in relative risk (RR) of 0.36% (95% CI: 0.12, 0.61). Conversely, when temperatures drop below 2.9°C, there is a marked reduction in relative risk, recorded at –0.63% (95% CI: −0.94, −0.33). This pattern suggests that maximum temperature may significantly influence negative sentiments risk, underscoring the importance of temperature in influencing emotions.

### Pollen exposure and negative sentiment risk

**Figure 2:**
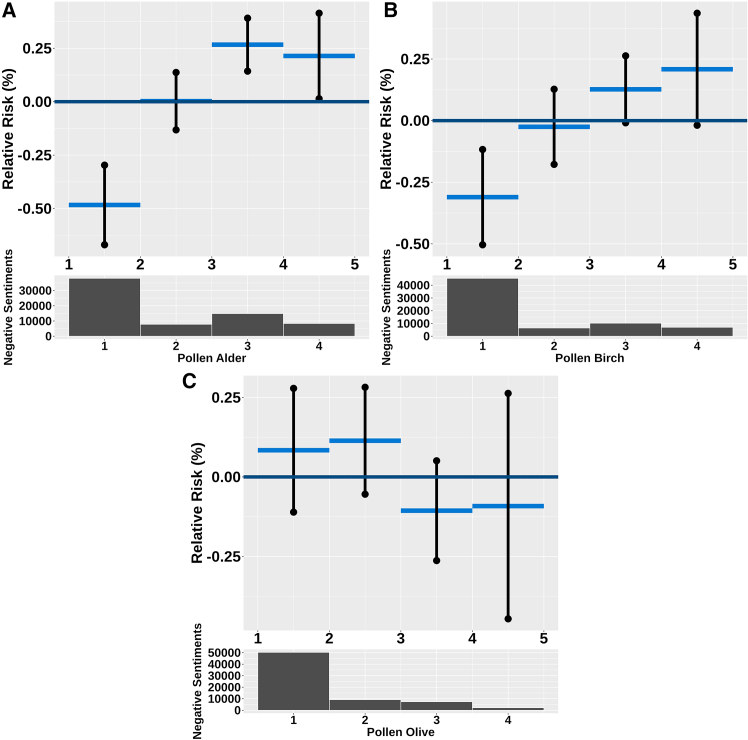
The non-linear effect of weekly alder, birch, and olive pollen counts (dark blue graph) has been analyzed by categorizing the weekly pollen in each region into four levels: low, mid, average, and high pollen counts The pollen alder categories are below 0.000512 grains/m^3^, from 0.000512 grains/m^3^ to 0.262 grains/m^3^, from 0.262 grains/m^3^ to 135 grains/m^3^, and above 135 grains/m^3^. The pollen birch categories are below 0.000530 grains/m^3^, from 0.000530 grains/m^3^ to 0.281 grains/m^3^, from 0.281 grains/m^3^ to 149 grains/m^3^, and above 149 grains/m^3^. The pollen olive categories are below 0.000393 grains/m^3^, from 0.000393 grains/m^3^ to 0.155 grains/m^3^, from 0.155 grains/m^3^ to 61.0 grains/m^3^, and above 61.0 grains/m^3^. The dark graph representing the upper and lower bounds shows that the effect is significant for alder and birch pollen. These upper and lower bounds are 95% significant bounds. The dark histogram below shows the distribution of the negative sentiments in each of the four categories for the three pollen types.

For alder pollen, when pollen (Figure 2A) counts exceed 135 grains/m^3^, the relative risk of negative sentiments rises by up to 0.21% (95% CI: 0.01 to 0.41). Conversely, when pollen counts are low or near zero, the relative risk is reduced to −0.48% (95% CI: −0.67 to −0.30). This indicates that higher alder pollen counts are associated with increased negative sentiments, while lower counts may alleviate these sentiments. Similarly, for birch pollen (Figure 2B), a clear pattern emerges as birch pollen counts increase to more than 149 grains/m^3^, and the relative risk of negative sentiments also rises, reaching up to 0.21% (95% CI: −0.02 to 0.44). Conversely, the relative risk declines significantly as birch pollen counts decrease toward zero. Both findings for birch and alder pollen are statistically significant, supporting the conclusion that elevated pollen levels can adversely affect the negative sentiments risk. In contrast to alder and birch pollen, our analysis of olive (Figure 2C) pollen showed that where the count is close to zero, the change in RR was 0.08% (95% CI: −0.11 to 0.28). For olive pollen levels above 61, the change in RR was −0.09% (95% CI: −0.45 to 0.26), revealing a reverse pattern concerning negative sentiments. Specifically, as olive pollen counts increased, the relative risk of negative sentiments decreased. However, this finding was not statistically significant, indicating that while there may be a relationship, the evidence is insufficient to draw definitive conclusions about the impact of olive pollen on negative sentiments.

### Heat-attributable mortality and West Nile virus incidence

**Figure 3:**
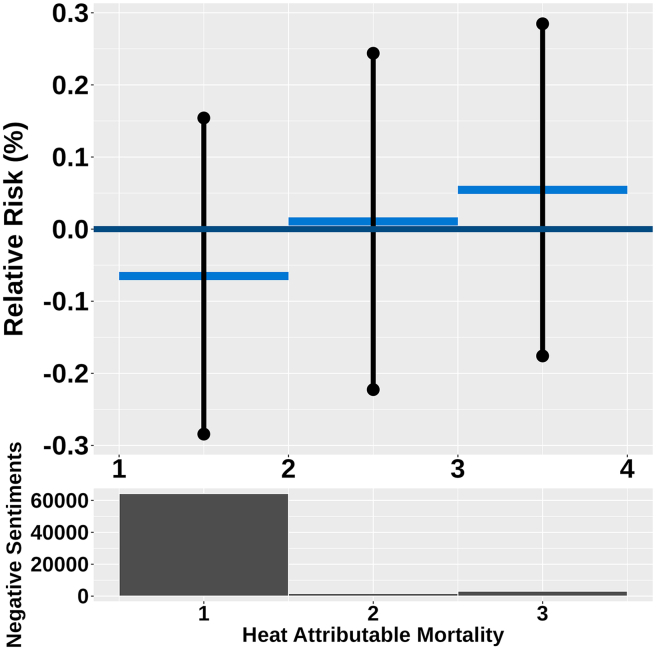
The non-linear effect of weekly heat-attributable mortality (dark blue graph) has been analyzed by categorizing the weekly heat-attributable mortality in each region into three levels: low, mid, and high mortality due to heat The heat-attributable mortality categories are below 0.00000168, from 0.00000168 to 0.382, and above 0.383. The dark graph representing the upper and lower bounds shows that the effect is insignificant for low, mid, and high mortality levels. These upper and lower bounds are 95% significant bounds. The dark histogram below shows the distribution of the negative sentiments in each of the three categories.

The analysis of heat-attributable mortality at the lowest category, the change in RR was −0.07 (95% CI: −0.28 to 0.15). For the middle category, the change in RR was 0.01% (95% CI: −0.22 to 0.24). At the highest category, the change in RR was 0.05% (95% CI: −0.18 to 0.29) (Figure 3), indicating that the contribution to relative risk from heat-attributable mortality values was above zero; however, these findings were not statistically significant. This suggests that while there may be an association between heat-related mortality exposure and risk of negative sentiment, the evidence is insufficient to establish a definitive link. Similarly, the contribution to relative risk for West Nile incidence, in areas with zero cases, the change in RR was −0.05% (95% CI: −0.63 to 0.53). In areas with at least one case, the change in RR was 0.05% (95% CI: −0·53 to 0·64) (Figure S3) was observed to be around zero, indicating no meaningful association with negative sentiments, reinforcing the idea that the impact of West Nile incidence on negative sentiments risk.

### Drought conditions and negative sentiment risk

**Figure 4:**
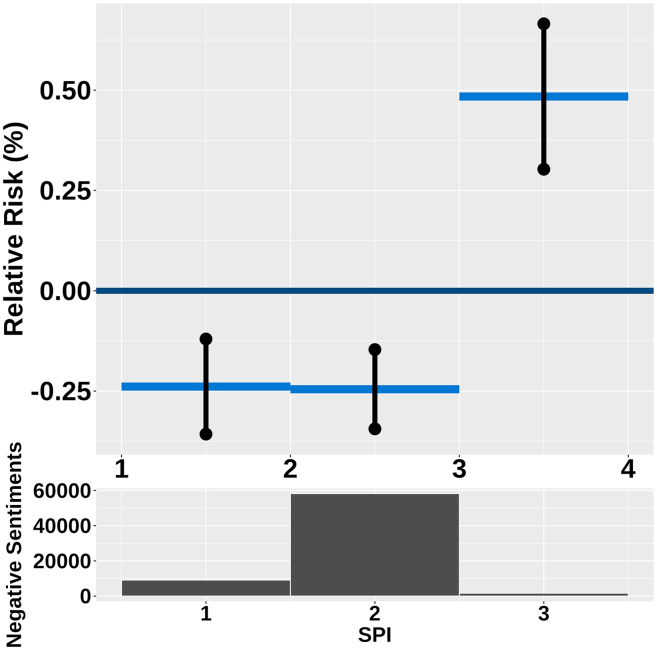
The non-linear effect of weekly drought (dark blue graph) has been analyzed by categorizing the weekly drought index in each region into three levels: rainfall, mid, and extreme drought The drought categories are as follows: below −3.72 indicates extreme drought (SPI unit), from −3.71 to 1.22 represents moderate drought, and above 1.22 signifies conditions where rainfall occurs. The dark graph representing the upper and lower bounds shows that the effect is significant for the presence of rainfall, and mid, and extreme drought levels. These upper and lower bounds are 95% significant bounds. The dark histogram below shows the distribution of the negative sentiments in each of the three categories.

The standardized precipitation index (SPI) analysis revealed notable associations with the risk of negative sentiments. When there is an excess of rainfall or moderate drought conditions, the relative risk of negative sentiments remains low (Figure 4) at −0.24% (95% CI: −0.36 to −0.12). This suggests that these conditions do not significantly contribute to negative emotional outcomes. However, as the SPI decreases and falls below −3.72 in extreme drought conditions, the relative risk of negative sentiments increases significantly, reaching up to 0.49% (95% CI: 0.30 to 0.67). This finding indicates that severe drought conditions are associated with a heightened risk of negative sentiments, emphasizing the potential impact of prolonged dry periods on negative sentiments risk.

## Discussion

Our results highlight the significant associations between various climate health indicators and the risk of negative sentiments. Notably, we found that increases in maximum temperature, alder pollen, birch pollen, and drought index correspond to a heightened relative risk of negative sentiments. Conversely, decreasing these factors leads to a statistically significant reduction in the relative risk of negative sentiments. Other situations, such as heat-attributable mortality, olive pollen, WNV incidence did not significantly correlate with risk of negative sentiment. This suggests that while certain environmental stressors may exhibit visible impacts on emotions, others may not.

The increase in maximum temperature is particularly noteworthy, as higher temperatures can lead to increased discomfort and heat stress, all of which may contribute to heightened negative sentiments.^22^ Similarly, the increase in pollen levels may trigger severe allergic reactions,^23^ which could increase the negative emotions. The same study showed that when alder pollen counts exceeded 45 grains/m^3^, there was a corresponding rise in drug consumption. Our research observed that when pollen counts surpassed 130 grains/m^3^, there was a significant association with an increased risk of negative sentiments. Extreme climatic events like drought play a critical role as they can also create other stressors related to water scarcity, agricultural impacts, and economic uncertainty.^24^ The psychological effects of drought—such as worry about resource availability and uncertainty about the future—can amplify feelings of helplessness and anxiety, leading to increased negative sentiments. It is worth mentioning that some parameters did not show associations with risk of negative sentiment. This suggests that while certain environmental stressors impact negative sentiments risk, others may not exert the same influence or may interact in more complex ways that warrant further investigation, as the association was not clear in this study. The lack of association between heat-related mortality, WNV incidence, and negative sentiments could be due to several factors. Heat-related mortality often disproportionately affects older adults, who may not be as active on the X platform or other sentiment-measuring platforms, leading to underrepresentation of their experiences. Similarly, WNV outbreaks are often localized and may occur in less densely populated areas, limiting public awareness and sentiment expression. Additionally, the general population might not perceive either heat or WNV as immediate threats, reducing the likelihood of strong emotional reactions. These factors suggest a potential mismatch between the affected populations and those generating sentiment data.

In comparison to the existing literature, our results indicate that an increase in maximum temperature was associated with an increase in risk of negative sentiment which aligns with previous studies conducted globally which found that extreme weather events worsen the sentiments.^9^^,^^25^ In our study, we found that lower temperature below 2.96°C was associated with a reduction in the relative risk of negative sentiments which is consistent with the findings of another study^17^ which noted that extremely low temperature correlates with lower negative sentiments. However, our findings diverge from the other findings of the same study,^17^ which suggests that maximum temperature decreases sentiments overall. To our knowledge, no previous study has correlated standardized precipitation index (drought) to negative sentiments; in our study, we found that extreme drought was associated with an increase in risk of negative sentiment. This aligns with the Lancet countdown 2022^16^ findings, showing that heatwaves and excessive precipitation increase negative sentiment and decreased positive sentiment in online expressions. Our study is the first to link pollen exposure to an increased risk of negative sentiments. However, a previous European indicator^21^ highlighted that pollen allergens are associated with conditions such as allergic rhinitis, allergic rhinoconjunctivitis, and bronchial asthma, affecting an estimated 40% of the European population. The high prevalence of these allergic diseases may contribute to our findings’ heightened relative risk of negative sentiments. Climate change and land-use modifications are increasing pollen quantities, prolonging pollen seasons, and enhancing allergenicity.^26^ Short-term increases in ambient pollen have been associated with higher rates of general practice consultations for allergic conditions, increased hospital admissions for asthma, and chronic obstructive pulmonary disease, and severe events such as thunderstorm asthma, particularly among sensitized individuals.^27^^,^^28^ Given projections of further climate-driven increases in pollen exposure, the health burden of allergic diseases is expected to rise, underscoring the importance of monitoring and mitigating these effects. We included parameters such as heat mortality in our model, recognizing that mortality can significantly impact individual well-being. Additionally, we incorporated WNV incidence, given the belief that infectious diseases^29^ during outbreaks and pandemics can affect mental health. However, our analysis did not reveal a clear association between West Nile incidence and negative sentiments, indicating that this relationship requires further investigation to understand better the potential impacts of infectious diseases on mental well-being.

This study offers several key implications for public health, policy, and future research. By integrating a comprehensive range of climate-induced stressors for the first time in our study, such as extreme climatic events (e.g., drought), climate-sensitive infectious disease (e.g., WNV incidence), shifts in pollen seasons, and heat-attributable mortality—along with the analysis of negative sentiments derived from the X platform, we introduced a novel, multifaceted approach on the impacts of climate health indicators on the sentiments of the individuals.

By leveraging the digital textual data from the X platform in real time and performing sentiment analysis as a proxy for subjective well-being, public health authorities can use this analysis to identify areas with emerging negative emotions related to climate-induced stressors. This allows for earlier interventions and improved communication strategies. The severe emotional response due to heightened environmental factors or diseases can be useful for targeted mental health resources and support during these events.

Second, policymakers and health organizations can gain insights into how the public perceives and reacts to climate-related risks in real time. This sentiment data can be used alongside traditional health indicators to understand the level of awareness and preparedness among communities due to climate stressors. These data will be used to inform awareness campaigns or educational initiatives about public understanding and preparedness, ensuring that the community is well-equipped to respond to climate-induced health risks.

This study underscores the need for more holistic climate change adaptation measures, as traditional studies focusing on one environmental factor might overlook other significant factors, such as changes in pollen seasons or disease vectors, contributing to public health challenges. By examining these factors across both temporal and spatial scales, we gain a more comprehensive understanding of how different regions and populations are impacted at various times. Social media sentiment data add a layer of understanding, offering a dynamic and up-to-date method for tracking public reactions to these stressors and aiding policymakers to implement more timely responses. Furthermore, the X platform’s large and diverse user base in Europe enables the capture of diverse voices, contributing to a more inclusive understanding of how various demographic groups perceive and respond to climate-related risks. As such, social media data provide a valuable supplement to conventional health indicators, offering a proactive tool for managing public health in the face of evolving climate and global health challenges.

This study highlights how climate-related stressors, such as maximum temperatures, drought, and pollen levels, can negatively affect negative sentiments and might proxy for well-being. The findings suggest that online sentiment data may serve as a useful indicator of climate-induced stress in populations, reflecting individuals’ challenges in adapting to environmental changes.

### Limitations of the study

This study faces several limitations related to data sources and analysis methods. First, despite the analysis being based on around 300 million geotagged tweets from 11 countries, our analysis is based on geotagged tweets, which constitute a very small proportion of all tweets and represent a non-random, demographically skewed subset of the population. Twitter users are typically younger, more urban, and more technologically engaged than the general population, and geotagged content further narrows this subset. Unfortunately, as our dataset lacks explicit demographic attributes (e.g., age, gender, and urban/rural classification), we could not directly assess representativeness against Eurostat population distributions. Therefore, our results should be interpreted as reflecting the expressed sentiments of active Twitter users rather than the general population. Additionally, while the LIWC22 sentiment analysis tool is highly validated and was chosen for its precision, it is not entirely accurate with challenges such as detecting sarcasm, code-switching, regional dialects, and cultural nuances potentially leading to misclassification of emotions. Furthermore, the study’s geographical scope is limited by the availability of social media data and supported languages, excluding some less connected regions. Additionally, while this study identifies correlations between climate stressors and sentiment shifts, establishing direct causality is challenging, as emotional responses could be influenced by other concurrent social, economic, or political factors. We considered same-week associations between exposures and expressed sentiments for comparability with previous studies, including our earlier analysis of climate and social media sentiment in Germany. However, emotional responses may occur in anticipation of or following a stressor. Future research should incorporate distributed-lag or cross-correlation models to better understand the timing and persistence of these effects. A further limitation is the sparsity of certain covariates, particularly heat-attributable mortality and WNV incidence. Although we addressed this by grouping exposures and applying a Bayesian hierarchical framework with partial pooling, the low counts may still limit statistical power. Future studies with larger datasets and alternative modeling approaches (e.g., zero-inflated or hurdle models) could provide additional insights.

### Future directions

One critical direction is the need for a unified sentiment analysis by leveraging AI advancements, particularly through deep-learning models like transformers. This would help build a more unified and scalable sentiment analysis tool that captures more accurate emotional expressions. This approach would significantly improve the robustness of sentiment detection in multilingual contexts by better understanding complex language structures and nuances, such as sarcasm or cultural variations in expression. A more comprehensive, multilingual sentiment analysis tool would significantly enhance the accuracy and inclusivity of future studies. Further research should also explore integrating multiple media channels, including social media platforms, news outlets, and other digital communication networks, to provide a more holistic understanding of public sentiment. In today’s interconnected world, these channels are crucial for obtaining real-time insights into public health research. Researchers can capture a broader spectrum of voices by leveraging data from various sources—beyond platforms like X (formerly Twitter), offering more comprehensive health insights and identifying emerging trends related to climate-induced stressors in real time. Future studies should be particularly mindful of the presence of outliers in social media data. Outliers can significantly affect the results of the outcome of interest, and researchers must exercise caution in both the analysis and interpretation of their findings. Additionally, a deep understanding of the nature of social media data, including its variability and potential biases, is crucial for drawing accurate and meaningful conclusions. It is also to continue incorporating cross-thematic topic analysis from multiple fields, such as epidemiology, public health, psychology, and environmental science. This integration will allow us to understand better how diverse stressors impact individual well-being. Future studies should investigate whether the observed stress and anxiety data related to climate stressors are real and have measurable psychological responses. This validation is crucial to distinguish real mental health impacts from perceived or transient sentiments.

Another important challenge for future research is ensuring the authenticity and reliability of social media signals. Automated accounts (bots) and coordinated disinformation campaigns can artificially amplify certain narratives, creating misleading sentiment trends. Developing and applying robust bot-detection algorithms and behavioral filters will be essential to minimize these biases. Furthermore, recent shifts in platform usage, with a decline in engagement on X and migration toward alternative platforms, may affect representativeness and continuity of digital health surveillance. Future studies should consider multi-platform integration and adaptive methods to address these evolving dynamics.

We acknowledge ongoing discussions about the ethics of repurposing social media content for research and surveillance, particularly regarding user consent and privacy expectations. Future public health applications should be guided by clear governance frameworks and ethical guidelines that balance potential societal benefits with respect for individual privacy.

## Resource availability

### Lead contact

Requests for further information and resources should be directed and will be fulfilled by the lead contact, Joacim Rocklöv (joacim.rocklov@umu.se).

### Materials availability

This study did not generate new unique materials.

### Data and code availability

• All data reported in this publication will be shared by the lead contact upon request.
• Code used in the analysis will be made publicly available immediately after publication through this link https://github.com/alahdalta1/Germany_Climate_Sentiment_Paper.git.
• Any additional information required to reanalyze the data reported in this article is available from the lead contact upon request.

## Acknowledgments

We acknowledge the support of the High-Performance Computing facility at the Interdisciplinary Center for Scientific Computing (IWR) for providing computational resources. We acknowledge that the present contribution is supported by the 10.13039/501100009318Helmholtz Association under the joint research school HIDSS4Health Helmholtz Information and Data Science School for Health. J.R., T.A.-A., and S.B. received funding from the 10.13039/100005156Alexander von Humboldt Foundation. The funding source has no involvement in the study design, data collection, analysis, interpretation, writing, or in the decision to submit the paper for publication.

## Author contributions

Conceptualization, T.A.-A., S.B., and J.R.; methodology, T.A.-A. and S.B.; software, T.A.-A. and S.B.; formal analysis, T.A.-A. and S.B.; investigation, T.A.-A.; resources, T.A.-A., B.A., and S.B.; data curation, B.A., T.A.-A., E.G., M.S., and J.B.; writing – original draft, T.A.-A. and S.B.; writing – review & editing, T.A.-A., S.B., B.A., M.G., E.G., M.S., J.B., T.B., M.R., and J.R.; visualization, T.A.-A. and J.R. ; supervision, M.G., T.B., and J.R.; project administration, T.A.-A; Funding Acquisition, J.R.

## Declaration of interests

The authors confirm that they have no conflicts of interest to disclose regarding this work.

## STAR★Methods

### Key resources table

**Table:** 

REAGENT or RESOURCE	SOURCE	IDENTIFIER
**Deposited data**		

ERA5 Climate Reanalysis Data	Copernicus Climate Data Store	https://cds.climate.copernicus.eu/
Pollen Reanalysis Data SILAM)	Finnish Meteorological Institute	https://silam.fmi.fi/
Heat-Attributable Mortality	EUROSTAT	https://ec.europa.eu/eurostat
West Nile Virus Incidence	European Center for Disease Prevention and Control (ECDC)	https://www.ecdc.europa.eu

**Software and algorithms**		

LIWC 22	LIWC Inc./Pennebaker et al.^30^	https://www.liwc.app/
R-INLA Package	Rue et al.^31^	https://www.r-inla.org/
genlasso Package (v1.6.1)	Tibshirani et al.^32^	https://cran.r-project.org/
R Statistical Software (v4.2.2)	R Foundation for Statistical Computing	https://www.r-project.org/
Python (v3.11.2)	Python Software Foundation	https://www.python.org/
Codes used in the analysis	Al-Ahdal et al.^14^	https://github.com/alahdalta1/Germany_Climate_Sentiment_Paper.git

### Experimental model and study participant details

• **Sample description**: This study analysed 462 million geotagged tweets posted by Twitter users across 11 European countries between January 1, 2015, and December 31, 2022.
• **Geographic coverage**: Austria, Belgium, France, Germany, Ireland, Italy, Netherlands, Portugal, Spain, Switzerland, and the United Kingdom, NUTS2 regions analysed.
• **Demographic limitations**: sex or gender, age, and other demographic characteristics of Twitter users were not available from the dataset and could not be analysed. This represents a limitation of the study.
• **Ethics statement**: The data used in this study was secondary data and deidentified so no ethical approval was needed to conduct this study.

### Method details

#### Data sources and harmonization

We integrated multiple datasets to support our analysis. Geo-located social media data was sourced from the Centre for Geographical Analysis, using tweets collected via the Twitter Streaming API with spatial coordinates and accuracy flags. We included only geotagged tweets posted between January 1, 2015, and December 31, 2022, in one of seven supported languages (English, German, French, Italian, Spanish, Portuguese, Dutch). Retweets, replies, and tweets without valid geolocation were excluded. Full preprocessing details are provided in the supplemental information. Climatic variables (e.g., mean, max, and min temperatures) were derived from the ERA5 dataset at the NUTS2 level, aggregated into 7-day averages. Pollen data covering alder, birch, and olive trees was obtained from a SILAM-driven reanalysis, providing weekly pollen levels from 2015 to 2022. Heat-attributable mortality estimates were based on EUROSTAT data (2015–2019) and modeled for 2015–2022. Additionally, West Nile virus infection data from the ECDC helped track its spatial and temporal distribution across Europe. Further details about data and processing in the supplemental information.

#### Sentiment analysis and preprocessing

The data is then prepared for sentiment analysis after a comprehensive preprocessing pipeline that removed emojis, hashtags, URLs, and non-alphabetic characters, during which we tested various sentiment analysis techniques, including the VADER,^11^ German sentiment model.^33^ Since our work spans multiple languages, we selected a method that could provide consistent and accurate sentiment measurements across different linguistic contexts. We used the LIWC22 (Linguistic Inquiry and Word Count), a highly validated tool that offers dictionaries tailored to multiple languages, ensuring a more reliable analysis. LIWC22^12^ operates using a lexicon of words and word stems linked to linguistic and psychological features, allowing for precisely measuring sentiments such as positive and negative emotions. While a full-scale multilingual validation was beyond the scope of this study, exploratory checks on random tweet samples suggested that LIWC22 classifications were consistent and aligned with expectations, supporting its suitability for large-scale analysis. This tool was adopted for the final analysis to ensure comprehensive and cross-linguistic sentiment assessment. the approach has been previously published elsewhere.^12^

#### Statistical analysis (modeling)

We employed a Poisson generalized additive model (GAM) to analyze the count of negative tweets in each region i *and* week t. This model is suitable for counting data, allowing for non-linear relationships among the covariates.

The model can be expressed as:

$$Y_{\left(i,t\right)}∼Poisson\left(\mu_{\left(i,t\right)}\right)$$

Where $Y_{\left(i,t\right)}$ represents the outcome in our study is the number of tweets with negative sentiments at region i and week t.

The mean function $\mu_{\left(i,t\right)}$ is defined by the following components:

1. $\theta_{tot}$: It represents the Overall Proportion of Tweets with predominance of negative sentiment expression: this parameter indicates the ratio of tweets with the predominance of negative sentiment expression to total tweets across the whole dataset.
2. $CT_{\left(i,t\right)}$: The total count of tweets in region i at week t.
3. $RR_{\left(i,t\right)}$: The relative risk of negative tweets in region i at week t, compared to the overall pattern.

The formula of the mean function can be represented as follows:

$$\mu_{\left(i,t\right)}=\theta_{tot}.CT_{\left(i,t\right)}.RR_{\left(i,t\right)}$$

We utilized a logarithmic link function to model the relative risk:

$$\log\left(RR_{\left(i,t\right)}\right)=S-T\;field_{\left(i,t\right)}+\sum_{h}f_{h}\left(covariat{e_{h}}_{,\left(i,t\right)}\right)+fused\;lasso_{c\left(i\right),I\left(t\right)}$$

Here, the $S-Tfield_{\left(i,t\right)}$ represents the spatiotemporal field which captures both regional and seasonal variations, while the random effects $f_{h}$ Fitted to covariates account for additional factors, such as climatic factors, pollen concentration, heat-attributable mortality, and WNV incidence in our study. $covariat{e_{h}}_{,\left(i,t\right)}$ denotes the value of covariate h in region i at week t. The last term, $fused\;lasso_{\left(c\left(i\right),I\left(t\right)\right)}$, denotes a random effect fitted to fused lasso coefficients Fused lasso is a regularization technique that encourages sparsity^32^ in the model by minimizing the number of non-zero coefficients. Essentially, this approach penalizes including many non-zero coefficients, ensuring that only the most significant trends in the data are captured. The fused lasso imposes sparsity on the coefficient values and penalizes differences between successive coefficients in the time series. This feature aims to minimize the number of abrupt changes or “jumps” in the coefficients over time. By incorporating the fused lasso, our model gains enhanced flexibility, allowing it to effectively capture significant fluctuations in the count of negative sentiments of the tweets between weeks. This capability is particularly beneficial for understanding dynamic patterns in the data, enabling a more nuanced analysis of the relationship between climate-induced climate-sensitive stressors and public sentiments. Specifically, fused lasso was applied separately to each of the 11 countries to estimate country-specific coefficients. The fitted fused lasso coefficients for each country c is a piece-wise constant time-series. This defines intervals of consecutive weeks t that have constant fused lasso coefficients. Model eq. (1) includes a fused lasso random effect for each country c, indexed by these intervals. With c(i) the country that region i belongs to, and I(t) the fused lasso-interval that week t belongs to, $fused\;lasso_{c\left(i\right),I\left(t\right)}$ denotes the corresponding random effect.

To account for both spatial and temporal dependencies in the data, we modeled the spatial component as independent and identically distributed (iid), assuming no spatial correlation between locations. We used an autoregressive (AR1) process for the temporal component to capture the temporal dependencies, allowing values in adjacent time periods to be more closely related. This approach treats the data as spatially independent but temporally dependent, reflecting the correlation between observations over time while assuming no spatial correlation across geographic locations.

We categorized several covariates into discrete levels to enhance the analysis of their impacts on the negative sentiments. Specifically, we grouped the climatic variables, pollen counts, West Nile virus incidence, and heat-attributable mortality into three to four distinct levels (Table S4) based on their observed effects. This stratification allows for a more nuanced understanding of how variations in these factors influence the count of negative tweets, facilitating a clearer interpretation of the relationships between climate-induced sensitive stressors and negative sentiment.

To assess multicollinearity among climatic and environmental covariates, we computed pairwise correlations (Figure S1). Temperature metrics exhibited strong correlations (|r| > 0.9); therefore, we retained maximum temperature (Tmax) as the representative heat metric to reduce redundancy. All selected predictors, including Tmax, drought index (SPI), solar radiation, cloud fraction, pollen, and health-related variables, were modeled jointly in a Bayesian spatio-temporal framework. To stabilize the estimated relative risks, we applied a fused lasso penalty that enforces temporal smoothness by shrinking abrupt fluctuations across consecutive weeks, while preserving overall exposure–response patterns.

The model was fitted using the R-INLA package^34^ (version: 24·3·9), which is a model frame-work for efficient inference for complex hierarchical models. The fused lasso was done by using the package “genlasso” (version: 1·6·1). The entire data processing workflow was carried out using Python (version: 3·11·2) and R (version: 4·2·2) on the IWR Quadxeon servers. The processing power of the Quadxeon servers allowed us to perform large-scale computations efficiently, ensuring quick data turnaround even for complex tasks.

Inference was conducted under a Bayesian framework using Integrated Nested Laplace Approximation (INLA) for approximate posterior estimation. We report posterior means of relative risk (RR) with 95% Bayesian confidence intervals (CI) derived from the posterior marginal distributions. Model fit was assessed using Pearson residual plots to detect potential systematic departures from model assumptions. These plots indicated residuals centered around zero without strong temporal trends.

### Quantification and statistical analysis

#### Sample size and reporting

This study analyzed *n* = 462 million tweets, where n represents individual geotagged social media posts from Twitter users. Statistical details including exact *n* values, test statistics, relative risks, and 95% Bayesian confidence intervals are reported throughout the results section and in Figure legends. Statistical measures reported include posterior means, 95% Bayesian confidence intervals (CI), relative risks (RR).

#### Software and approach

The INLA (Integrated Nested Laplace Approximation) method was implemented using R version 4.0.3 (R Foundation for Statistical Computing). The INLA package (version 20.01) was used to run the Bayesian model and perform statistical inference. The model was run on the IWR high-performance computing servers.

Python: Data cleaning and preprocessing of the textual data were carried out using Python version 3.8.5. Various Python libraries (e.g., pandas, numpy, and regex) were used to preprocess and organize the textual data before analysis.

Computational Resources: The data was processed and analyzed using IWR high-performance computing servers, which provided the necessary computational power for running the model and handling the large datasets involved in this study.

#### Statistical method

We used a Poisson generalized additive model (GAM) to model the relation between negative sentiments and climatic health factors.

#### Imaging

All figures in the paper were created using RStudio, with resolution adjustments made in Adobe Photoshop. The Graphical Abstract was designed using the free version of BioRender.
