# Supplementary material for: The association of climate-induced stressors on risk of negative sentiment: An analysis from 462 million geotagged tweets in Europe
Source: iScience. 2025 Nov 3;28(12):113933. doi: 10.1016/j.isci.2025.113933 (PMC12670924; doi:10.1016/j.isci.2025.113933)
Supplement: Document S1. Figures S1–S4, Tables S1–S6, and Methods S1 [file mmc1.pdf]

iScience, Volume *28*

Supplemental information

The association of climate-induced stressors on risk of negative sentiment: An analysis from 462 million geotagged tweets in Europe

Tareq Al-Ahdal, Sandra Barman, Barrak Alahmad, Stella Dafka, Elisa Gallo, Joan Ballester, Mikhail Sofiev, Marina Romanello, Till Bärnighausen, Michael Gertz, and Joacim Rocklöv

Supplementary Material for the Paper the Association of Climate-Induced Stressors on Risk of Negative Sentiment: An Analysis from 462 million Geotagged Tweets in Europe

Methods S1

Supplementary Tables S1-S6

Supplementary Figures S1-S4

References

Methods S1. Detailed procedures for data collection, processing, harmonization, and statistical modeling

Data Sources, Collection and Processing

Social Media X Platform Data

We utilized the Centre for Geographical Analysis (CGA) dataset [1], this comprehensive dataset includes geo-located tweets harvested using the Twitter Streaming API, which captures tweets with spatial attributes (Coordinates and Places). Each tweet is assigned latitude and longitude, derived from GPS data or calculated centroids of bounding boxes when GPS is unavailable. A GPS flag indicates the source of the coordinates, while a spatial error estimate helps interpret their accuracy.

Data Collection and Preprocessing

First, we collected all tweets from Europe by setting the region's maximum and minimum latitude and longitude boundaries during the study period, which spans from January 1, 2015, to the end of December 2022. The total digital cohort used in this study was 462 million tweets after removing the duplicates and selecting specific languages, approximately 300 million tweets.

Natural Language Processing

Natural Language Processing (NLP)[2] has become vital for extracting valuable insights from large-scale textual data, such as social media content. In this study, we employed scalable and efficient NLP techniques to handle the extensive volume of tweets collected over eight years. By using Regex (v2023.10.3), we ensured rigorous data cleaning, removing irrelevant elements for the objective of this study like hashtags, user mentions, and URLs. This preprocessing step helped eliminate noise, allowing for more accurate sentiment extraction.

We leveraged NLTK (v3.8.1) for tokenization and stop word removal, which ensured the text was refined to focus on meaningful linguistic patterns. To adapt to the specific characteristics of social media data, we customized the tokenization process and incorporated spaCY (v3.7.2) and TextBlob (v0.17.1) to structure further and enrich the textual data. This combination of tools enabled the efficient preparation of the tweets for analysis.

Country Selection

In selecting countries for our analysis, we focused on the languages available in LIWC22 to ensure robust and comparable sentiment scores between the different countries. The languages of the chosen countries—Austria, Belgium, France, Germany, Ireland, Italy, Netherlands, Portugal,

Spain, Switzerland, and the United Kingdom—are highly represented in the LIWC22 lexicon. In addition, these countries are located across different climatic zones in Europe[3], each experiencing distinct environmental challenges, such as rising temperatures, extreme weather events, and pollution. This geographical diversity allows for a comprehensive examination of how varied climate-related stressors impact emotional responses among different populations. We utilized several dictionaries, which are translations of the various LIWC dictionaries[4] developed over nearly 20 years. Specifically, we used the English LIWC-22 Dictionary, the DE-LIWC2015 Dictionary for German, the LIWC 2007 Dictionary for Spanish, the LIWC 2015 Dictionary for Brazilian Portuguese, the LIWC 2007 Dictionary for Italian, the LIWC 2007 Dictionary for French, and the LIWC 2015 Dictionary for Dutch.

Climate-Health hazard data

Climatic Data

The study employed climatic data from the ERA5 dataset[5], sourced from the European Centre for Medium-Range Weather Forecasts (ECMWF), to analyze various variables at the NUTS2. The final dataset included 7-day averages and accumulations of key climatic variables such as mean temperature, maximum temperature, minimum temperature.

Pollen Data

We obtained pollen data from a reanalysis dataset[6] containing pollen seasons for three major allergenic genera of trees in Europe: alder (Alnus), birch (Betula), and olive (Olea). This dataset, driven by the ERA5 meteorological reanalysis, utilizes the atmospheric composition model SILAM to predict flowering periods and pollen dispersion patterns. Our study specifically focused on the average weekly pollen data from 2015 to 2022.

Heat Attributable Mortality

Mortality data was utilized from EUROSTAT, covering the period from January 2015 to December 2019, to calibrate the epidemiological models. These models enabled the conversion of temperature and mortality time series from January 2015 to November 2022[7-9] into weekly heat-related mortality estimates for the years 2015 to 2022.

We obtained West Nile virus infection notifications from the European Centre for Disease Prevention and Control (ECDC).[10] This dataset provides crucial information for understanding the geographical distribution and incidence of West Nile virus infections across Europe.

Spatial and Temporal Data Alignment of data

Spatial aggregation is essential for associating the data with administrative regions, thereby enhancing the analysis of the sentiment outcome about location. In this study, we implemented a robust spatial aggregation technique using Python's Pandas (version: 1.5.3), GeoPandas (version: 0.12.2), and Shapely (version: 1.8.5) libraries to process our dataset effectively. Utilizing GeoPandas, we loaded a shapefile containing the geographical boundaries of NUTS (Nomenclature of Territorial Units for Statistics) regions. This shapefile provided the necessary framework for mapping the geographical points based on their coordinates. The data was aggregated both at NUTS3 and NUTS2 levels.

Temporal aggregation is vital for analyzing sentiment trends over time, allowing for a clearer understanding of emotional responses about specific periods. In this study, we implemented an effective temporal aggregation technique using the lubricate (version: 1.9.2) library in R to process our dataset. We extracted key temporal features such as year, month, and week from the timestamp data, facilitating the classification of tweets into distinct time intervals. The data was then aggregated at the weekly level, providing insights into sentiment patterns across different time frames.

The final dataset, covering a span of eight years from 2015 to 2022, was aggregated weekly at the NUTS3, and NUTS2 levels, providing insights into sentiment patterns across different time frames.

Data Harmonization

Data harmonization is crucial in ensuring that diverse datasets can be effectively integrated and analyzed within a unified framework. In this study, we utilized the Integrated Nested Laplace Approximation (INLA)[11] spatiotemporal model, which offers flexibility in handling various types of input data, including continuous climatic data and categorical sentiment data. This flexibility allows for developing a joint modeling framework that accommodates different data characteristics. To facilitate this integration, we discretized the independent variables to derive relevant covariates. A notable strength of the INLA framework is its ability to incorporate non-linear random effects, which enhances model performance and reduces dependence on the scale and normalization of covariates. While normalization is still important for categorizing the covariates, it is less critical than traditional modeling approaches such as linear or quadratic models. Moreover, careful aggregation of the tweet data is essential; having a relatively equal number of tweets across regions improves model stability. Although the model accounts for the number of tweets, achieving a balance in tweet distribution among regions contributes to more reliable performance. In this context, we observed better stability when analyzing data at the NUTS2 level compared to the NUTS3 level due to the larger number of tweets per region.

Data Privacy and Relevance

In our analysis of X data, we prioritized user privacy and adhered to best practices for data protection. To ensure confidentiality, we implemented deidentification measures for usernames and handles, effectively anonymizing any personal information that could potentially identify individual users.

Our focus was primarily on the textual content of the tweets, as well as their associated geographical information, specifically latitude and longitude. By concentrating on these data points, we aimed to minimize privacy risks while still extracting valuable insights from the dataset.

To facilitate sentiment analysis, we divided the data by language. This approach allowed us to apply LIWC (Linguistic Inquiry and Word Count) dictionaries specific to each language, enhancing the accuracy and relevance of our sentiment analysis. Each language dataset was aggregated separately, allowing for a nuanced examination of sentiment across different linguistic groups. After performing sentiment analysis on the individual language datasets, we aggregated the results to create a comprehensive overview of the entire dataset. This process required substantial computational resources, as it involved processing and analyzing large volumes of textual data while maintaining data integrity and confidentiality.

LIWC22

The LIWC (Linguistic Inquiry and Word Count) software operates independently of traditional Natural Language Processing (NLP) libraries. It utilizes its own dictionaries and algorithms to analyze text by categorizing words into various psychological and linguistic dimensions. While LIWC can complement NLP tasks, it does not require NLP frameworks for its functionality. Refer to the Development and Psychometric Properties of LIWC-22.[12]

LIWC (Linguistic Inquiry and Word Count) is a text analysis tool that assesses psychological and emotional states by analyzing word usage patterns. LIWC-22, the latest version, uses updated dictionaries for every individual language to categorize words into psychologically meaningful categories, including "positive emotion" (pos emo) and "negative emotion" (neg emo).

Pollens and Climatic Data processing

We utilized R programming language to process spatial weather data from NetCDF files, focusing on NUTS3 regions defined in a shapefile. A shapefile containing NUTS 3 regions was read and filtered, followed by generating a list of relevant NetCDF files. Each file was read, reprojected, and cropped to the extent of the shapefile, with mean values extracted for each district using a spatial extraction method. Finally, the extracted data was organized by date and saved as CSV files for further analysis.

Categorization of the predictors

The binning process applied to the predictors in our dataset effectively transformed continuous data into discrete categories. This approach not only simplified data analysis but also improved the interpretability of the results. By carefully considering the distribution of predictor values and making thoughtful adjustments to the binning structure, we were able to create a framework that facilitates a deeper understanding of the covariates and their implications on the outcome.

Correlation and Descriptive Data Graphics

We performed a correlation analysis to assess multicollinearity among the predictors in our model. The resulting correlation matrix (Figure S1) indicates several correlations among predictors. Notably, temperature variables exhibited a strong correlation with each other ($|r| > 0.9$), raising concerns about potential multicollinearity. We also observed a moderate negative correlation between cloud fraction, relative humidity, and temperature, while solar radiation showed a positive correlation with temperature. In contrast, pollen parameters displayed negligible correlation with other variables, suggesting that they provide independent information in the model.

Based on these findings, we retained **maximum temperature (Tmax)** as the representative temperature metric to minimize redundancy among highly correlated thermal variables. All predictors, including Tmax, SPI, solar radiation, cloud fraction, air quality, and pollen variables, were modeled jointly in a **penalized regression framework (fused lasso)**. This approach mitigates the influence of multicollinearity by shrinking less informative or redundant covariates toward zero, allowing the model to capture only the most relevant signals.

To complement the correlation analysis, we constructed a conceptual graph (Figure S2) to visually represent the relationships among selected predictors. The graph illustrates directional associations, such as the positive link between temperature and solar radiation and the negative association between cloud fraction, relative humidity, and temperature. Pollen variables are highlighted in gray, emphasizing their independence from climatic variables. This visualization, combined with penalization in the modeling strategy, ensured that we accounted for interdependencies while minimizing the risk of collinearity.

Model Specifications

To evaluate the role of spatial correlation, We fitted six alternative Poisson log-linear models (INLA) for each year (2015–2022) to assess the contribution of spatial structure and time Table S6:

- **Model 0:** spatial random effect **(iid)**; no covariates.
- **Model 1:** spatial random effect **(iid)** + covariates (**β**).
- **Model 2:** spatial random effect **(BYM2)**; no covariates.
- **Model 3:** spatial random effect **(BYM2)** + covariates (**β**).
- **Model 4: spatio-temporal random effect (iid × AR(1))** + covariates (**β**).
- **Model 5: spatio-temporal random effect (BYM2 × AR(1))** + covariates (**β**).

We compared models using Deviance Information Criterion (DIC), Widely Applicable Information Criterion (WAIC), and Conditional Predictive Ordinate (CPO).

Supplementary Tables

Supplementary Table S1: Language-wise distribution of tweets analyzed in the study, including total counts and percentages.

Language	Total Tweets	Percentage (%)
German	22,577,028	4.88
Portuguese	19,447,730	4.21
Dutch	21,364,420	4.62
Italian	33,849,115	7.32
French	57,473,395	12.43
Spanish	87,198,377	18.86
English	220,554,873	47.69
**Total**	**462,464,938**	**100**

Supplementary Table S2: Annual counts of tweets classified with negative sentiment during the study period

Year	Negative Sentiments Count
2015	2,122,222
2016	3,846,716
2017	6,685,688
2018	10,110,904
2019	18,899,850
2020	23,676,917
2021	14,471,659
2022	16,690,019

Supplementary Table S3: Category-specific relative risks (RR), percentage change in RR, and 95% confidence intervals for each exposure across categorical bins

Parameter	Bin	RR	% Change in RR	95% CI (Lower–Upper)
**SPI**	1	0.997615	–0.2385	–0.356635 – –0.120182
	2	0.997549	–0.2451	–0.343586 – –0.146499
	3	1.004853	0.4853	0.303335 – 0.667654
**Maximum Temperature**	1	0.993656	–0.6344	–0.936868 – –0.331033
	2	1.000367	0.0367	–0.110602 – 0.184298
	3	1.002873	0.2378	0.091761 – 0.383982
	4	1.003629	0.3629	0.11973 – 0.606607
**Air Quality**	1	1.000036	0.0036	–0.202166 – 0.209825
	2	1.000182	0.0185	–0.173067 – 0.210443
	3	0.999773	–0.0227	–0.377411 – 0.334505
**Heat Attributable Mortality**	1	0.999349	–0.0651	–0.283917 – 0.154241
	2	1.000107	0.0107	–0.2223 – 0.244175
	3	1.000545	0.0545	–0.175714 – 0.285146
**WNV Incidence**	1	0.999463	–0.0537	–0.63217 – 0.528073
	2	1.000538	0.0538	–0.5253 – 0.636192
**Wind Speed**	1	1.000033	0.0033	–0.231277 – 0.238383
	2	0.999677	–0.0323	–0.252571 – 0.188493
	3	0.998252	–0.1748	–0.401843 – 0.052685
	4	1.002042	0.2042	–0.404247 – 0.816382
**Pollen Birch**	1	0.9969	–0.31	–0.502642 – –0.117027
	2	0.999749	–0.0251	–0.177494 – 0.127539
	3	1.001271	0.1271	–0.008895 – 0.263328
	4	1.002088	0.2088	–0.018823 – 0.436888
**Pollen Alder**	1	0.995179	–0.4821	–0.667301 – –0.296475
	2	1.000265	0.0265	–0.142102 – 0.194798
	3	1.002675	0.2671	0.142359 – 0.391593
	4	1.002142	0.2142	0.014009 – 0.4148
**Pollen Olive**	1	1.00084	0.084	–0.110602 – 0.278921
	2	1.001139	0.1139	–0.060749 – 0.287541
	3	0.99894	–0.106	–0.26265 – 0.05081
	4	0.999084	–0.0916	–0.445175 – 0.263147

Supplementary Table S4: sensitivity analysis of exposure bins: relative risks (RR) with 95% confidence intervals

Exposure	Bin	Mean (RR)	Lower 95% CI	Upper 95% CI
Temperature	1	0.9952	0.9935	0.9980
	2	1.0018	1.0001	1.0032
	3	1.0029	1.0009	1.0049
Alder pollen	1	0.9964	0.9950	0.9980
	2	1.0019	1.0009	1.0030
	3	1.0016	0.9999	1.0030
Birch pollen	1	0.9978	0.9960	0.9994
	2	1.0008	0.9994	1.0018
	3	1.0019	1.0000	1.0032

Supplementary Table S5: Bin definitions for categorized environmental variables.

Exposure	Bin	Min	Max
Tmax (°C)	1	-9.00	2.96
	2	2.96	14.9
	3	14.9	26.9
	4	26.9	38.8
Pollen Alder	1	0	0.000511
	2	0.000512	0.262
	3	0.262	134
	4	135	68846
Pollen Birch	1	0	0.000529
	2	0.000530	0.281
	3	0.281	149
	4	149	79232
Pollen Olive	1	0	0.000391
	2	0.000393	0.155
	3	0.155	60.9
	4	61.0	23969

Exposure	Bin	Min	Max
SPI	1	1.22	3.68
	2	-3.71	1.21
	3	-8.64	-3.72
Wind Speed (m/s)	1	0.121	2.50
	2	2.50	4.88
	3	4.88	7.26
	4	7.27	9.65
Air Quality (μg/m³)	1	1.15	15.1
	2	15.1	29.1
	3	29.1	57.1

Table S6. Model specifications and fit metrics by year (2015–2022).

model	dic	waic	cpo	year_idx
model0	123116.7	266375	8.365357	1
model1	59645.41	62189.39	3.806967	1
model2	123115.3	266373.4	8.365451	1
model3	59647.11	62191.09	3.807091	1
model4	51738.9	51889.04	3.331726	1
model5	51683.64	51797.99	3.32081	1
model0	79547.36	79945.62	4.621044	2
model1	71564.88	75329.37	4.181636	2
model2	79546.98	79945.23	4.621012	2
model3	71564.31	75331.02	4.181617	2
model4	65738.67	65937.73	3.835554	2
model5	65719.2	65902.86	3.834092	2
model0	87258.84	87772.87	5.045225	3
model1	82381.99	90450.56	4.800405	3
model2	87258.65	87773.77	5.045207	3
model3	82382.1	90439.01	4.800385	3
model4	75568.87	75868.64	4.4012	3
model5	75544.5	75836.71	4.398974	3
model0	376195.9	4813699	27.29039	4
model1	107070.2	135931.8	6.620341	4
model2	376192.8	4813578	27.29042	4
model3	107076.3	136058.9	6.620319	4
model4	78479.01	78336.43	4.789751	4
model5	78275.54	78004.32	4.749975	4

model0	116175.1	143074.1	6.833902	5
model1	101311.2	109808.2	5.969416	5
model2	116175.3	143069.4	6.833907	5
model3	101311.4	109806.8	5.969427	5
model4	87454.16	87941.19	5.238488	5
model5	87330.38	87725.44	5.220738	5
model0	129159.9	138442.6	7.49662	6
model1	105651.1	107797.2	6.140483	6
model2	129159.9	138441.2	7.496617	6
model3	105651.1	107795.8	6.140484	6
model4	90781.21	91166.22	5.375512	6
model5	90683.85	91002.51	5.358932	6
model0	110079.8	120913.3	6.704734	7
model1	98645.99	99970.82	5.891073	7
model2	110080.2	120904.4	6.704747	7
model3	98646.28	99976.38	5.8911	7
model4	84886.22	85284.41	5.172907	7
model5	84766.8	85088.95	5.15166	7
model0	119803.2	161071.5	7.23901	8
model1	107934.4	127797.3	6.570277	8
model2	119803	161079	7.239018	8
model3	107934.2	127800.6	6.570284	8
model4	87474.93	87673.88	5.34312	8
model5	87318.73	87409.5	5.312809	8

Supplementary Figures

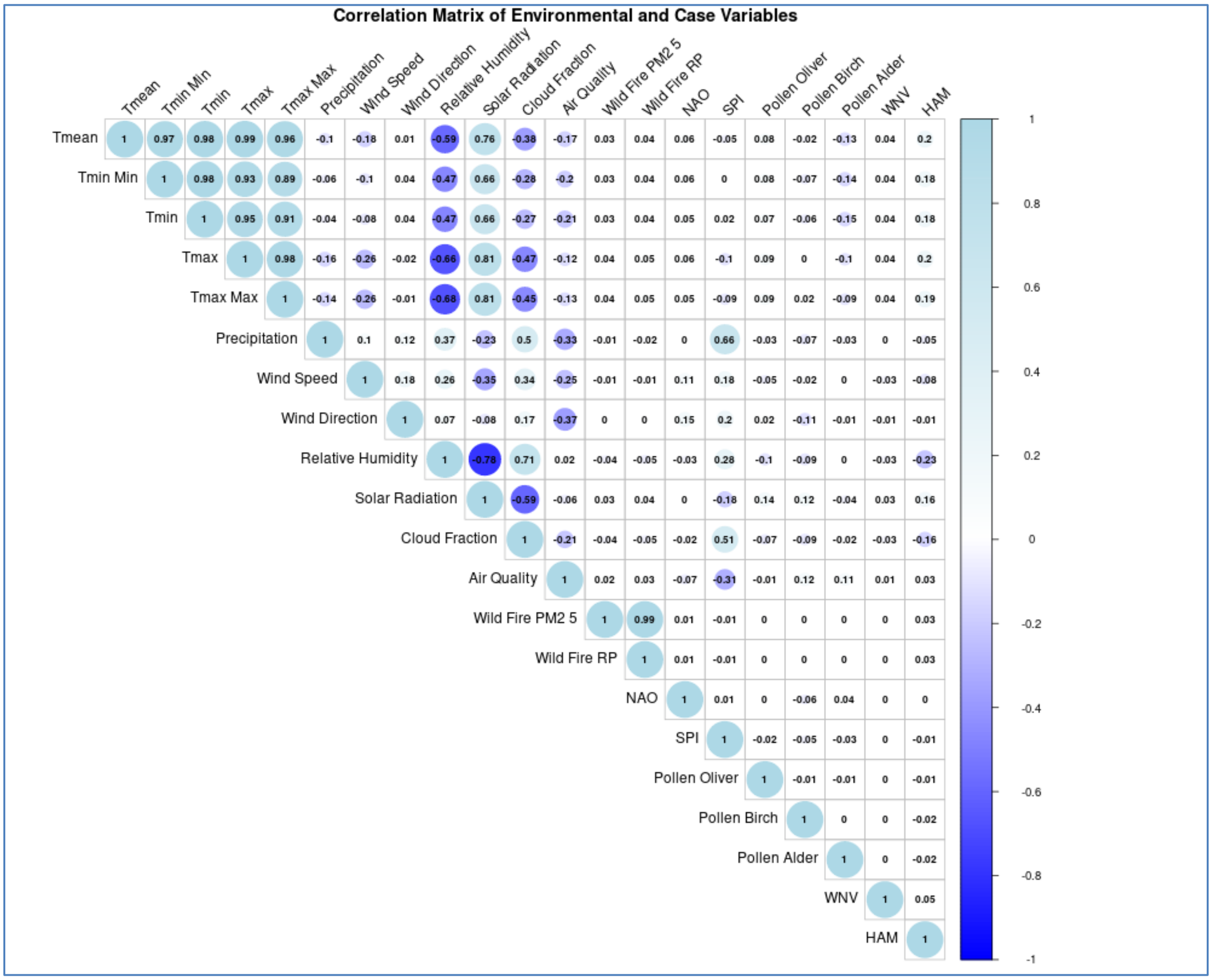

Figure S1: Correlation Analysis of the Predictors.

The color scale shows the direction, and the size of the circle shows the strength of the correlation, with higher positive values depicted by light aqua colors while lower negative values are represented by purple colors.

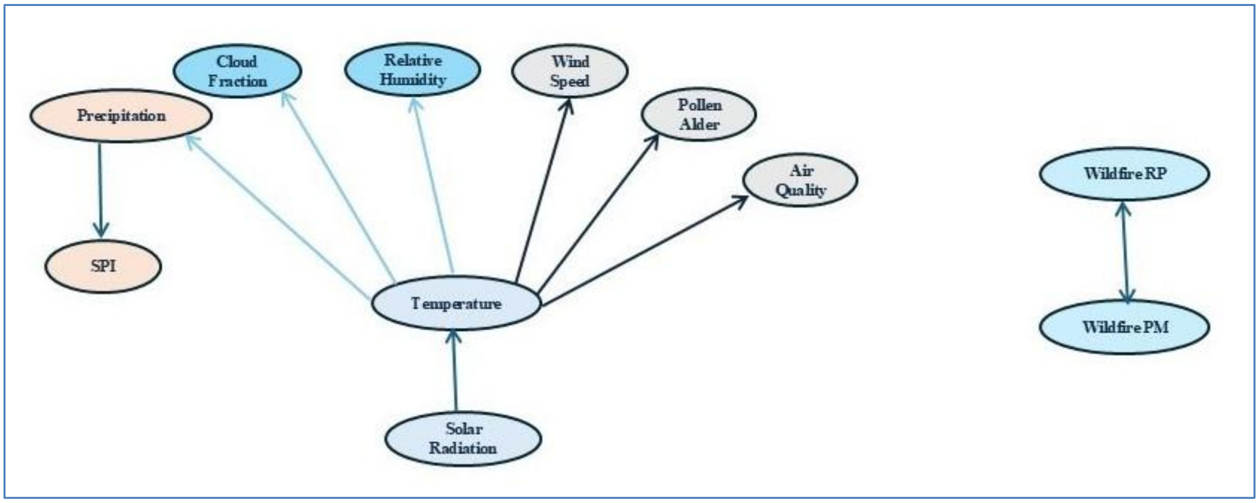

Figure S2: Representation of Predictor Relationships Using Correlation Matrix

This figure visualizes the correlations between selected predictors

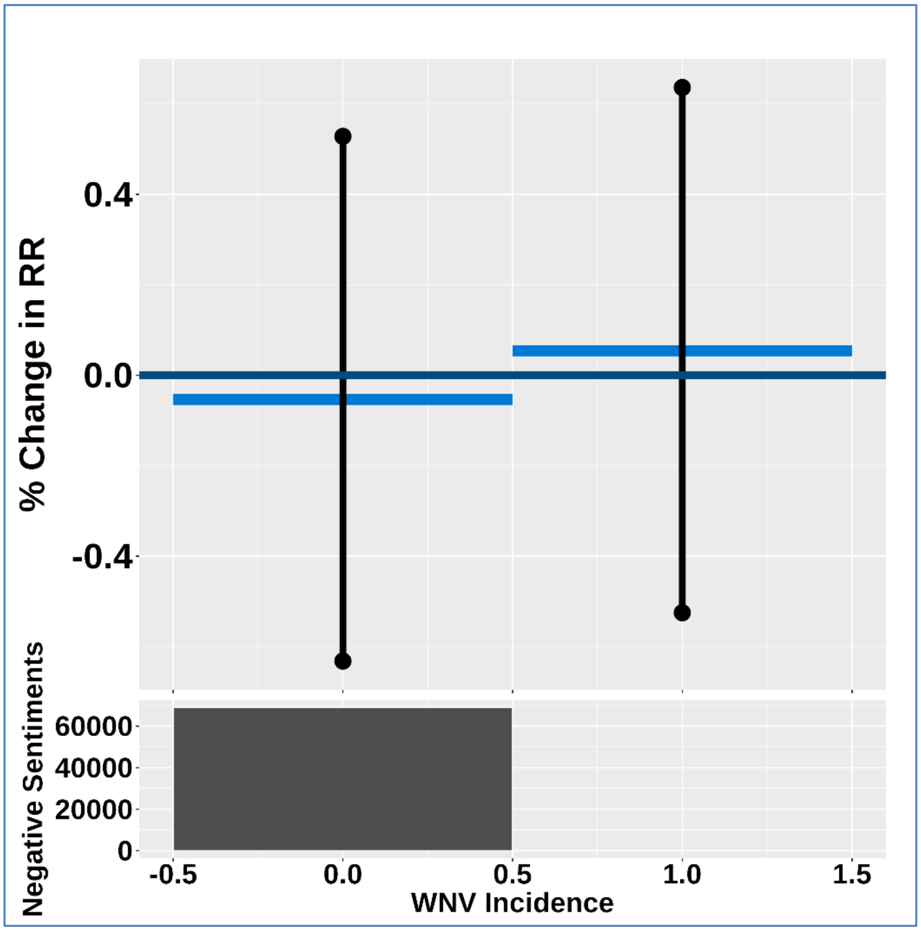

Figure S3: **The non-linear effect of weekly West Nile incidence (dark blue graph) has been analyzed by categorizing the weekly West Nile incidence in each region into two levels: Absence and presence of West Nile incidence.**

The West Nile incidence categories are 0 no incidence, or 1 **there is a West Nile case**. The dark graph representing the upper and lower bounds shows that the effect is insignificant for both categories. These upper and lower bounds are 95% significant bounds. The dark histogram below shows the distribution of the negative sentiments in each of the two categories.

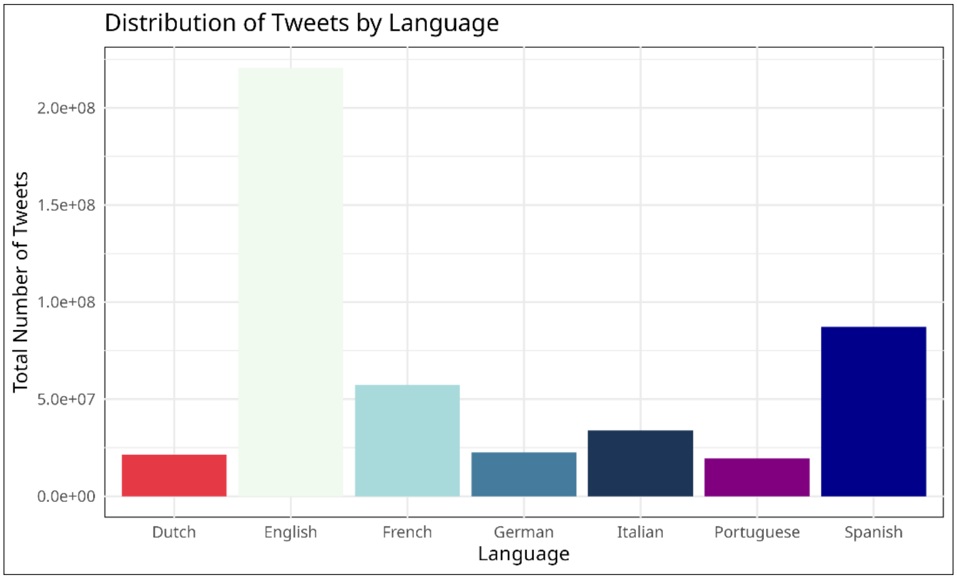

Supplementary Figure S4: Distribution of tweets by language. The figure illustrates that English tweets account for nearly half of the dataset (47.7%), followed by Spanish (18.9%) and French (12.4%). Other languages such as Italian, German, Dutch, and Portuguese represent smaller shares.

References

1. Analysis HUCfG. Geotweet Archive v2.0. In: Analysis CfG, editor.; 2025.
2. Sharma R, Agarwal P, Arya A. Natural language processing and big data: a strapping combination. New Trends and Applications in Internet of Things (IoT) and Big Data Analytics: Springer; 2022: 255-71.
3. Cui D, Liang S, Wang D. Observed and projected changes in global climate zones based on Köppen climate classification. *Wiley Interdisciplinary Reviews: Climate Change* 2021; **12**(3): e701.
4. LIWC. Linguistic Inquiry and Word Count. 2024. https://www.liwc.app/ (accessed 31.10.2024.
5. Service" CCC. ERA5 Climate Data. 2024.
6. Sofiev M, Palamarchuk J, Kouznetsov R, et al. European pollen reanalysis, 1980–2022, for alder, birch, and olive. *Scientific data* 2024; **11**(1): 1082.
7. van Daalen KR, Romanello M, Rocklöv J, et al. The 2022 Europe report of the Lancet Countdown on health and climate change: towards a climate resilient future. *The Lancet Public Health* 2022; **7**(11): e942-e65.
8. Ballester J, Quijal-Zamorano M, Méndez Turrubiates RF, et al. Heat-related mortality in Europe during the summer of 2022. *Nature medicine* 2023; **29**(7): 1857-66.
9. Ballester J, van Daalen KR, Chen Z-Y, et al. The effect of temporal data aggregation to assess the impact of changing temperatures in Europe: an epidemiological modelling study. *The Lancet Regional Health–Europe* 2024; **36**.
10. ECDC. European Centre for Disease Prevention and Control (ECDC)-West Nile Data. 2024. https://www.ecdc.europa.eu/en.
11. R-INLA. What is INLA? 2024. https://www.r-inla.org/what-is-inla31.10.2024).
12. Boyd RL, Ashokkumar A, Seraj S, Pennebaker JW. The development and psychometric properties of LIWC-22. *Austin, TX: University of Texas at Austin* 2022; **10**.
